# Resource Allocation for Cognitive LEO Satellite Systems: Facilitating IoT Communications

**DOI:** 10.3390/s23083875

**Published:** 2023-04-11

**Authors:** Bowen Cai, Qianqian Zhang, Jungang Ge, Weiliang Xie

**Affiliations:** 1 China Telecom Research Institute, Beijing 102209, China; caibw@chinatelecom.cn (B.C.); xiewl@chinatelecom.cn (W.X.); 2 China Telecom Research Institute, University of Electronic Science and Technology of China (UESTC), Chengdu 611731, China; gejungang@std.uestc.edu.cn

**Keywords:** Internet of Things (IoT), low earth orbit (LEO) satellite communication, cognitive radio, resource allocation

## Abstract

Due to the characteristics of global coverage, on-demand access, and large capacity, the low earth orbit (LEO) satellite communication (SatCom) has become one promising technology to support the Internet-of-Things (IoT). However, due to the scarcity of satellite spectrum and the high cost of designing satellites, it is difficult to launch a dedicated satellite for IoT communications. To facilitate IoT communications over LEO SatCom, in this paper, we propose the cognitive LEO satellite system, where the IoT users act as the secondary user to access the legacy LEO satellites and cognitively use the spectrum of the legacy LEO users. Due to the flexibility of code division multiple access (CDMA) in multiple access and the wide use of CDMA in LEO SatCom, we apply CDMA to support cognitive satellite IoT communications. For the cognitive LEO satellite system, we are interested in the achievable rate analysis and resource allocation. Specifically, considering the randomness of spreading codes, we use the random matrix theory to analyze the asymptotic signal-to-interference-plus-noise ratios (SINRs) and accordingly obtain the achievable rates for both legacy and IoT systems. The power of the legacy and IoT transmissions at the receiver are jointly allocated to maximize the sum rate of the IoT transmission subject to the legacy satellite system performance requirement and the maximum received power constraints. We prove that the sum rate of the IoT users is quasi-concave over the satellite terminal receive power, based on which the optimal receive powers for these two systems are derived. Finally, the resource allocation scheme proposed in this paper has been verified by extensive simulations.

## 1. Introduction

Internet-of-Things (IoT) applications, such as financial services, intelligent transportation, and remote sensing, are envisioned to be an important driving force of the smart society [1]. It can be seen, with the popularization of IoT applications, trillions of new devices will be connected to the networks for different application needs, such as smart wearable devices, smart homes, environmental monitoring, and so on [2]. A variety of IoT applications require IoT networks deployed remotely over large areas (e.g., environmental monitoring). However, due to the fact that the infrastructure construction is affected by the geographical environment, terrestrial wireless systems cannot achieve comprehensive coverage, especially in particular environments such as deserts, oceans, and forests [3]. As an extension and supplement of the terrestrial communication system, low earth orbit (LEO), medium earth orbit (MEO), and geostationary earth orbit (GEO) satellites can be used in areas that are not covered by terrestrial networks [4]. For years, industry and academia have focused on the GEO satellites to provide Internet access. But these high-orbiting satellites face significant challenges in providing services. For instance, signals need to span a distance of 36,000 km from Earth to the satellite’s return, and time delays often exceed 600 ms, which cannot meet many existing service demands [5]. In contrast, the LEO satellite systems between 160 and 2000 km can provide low-latency, high-speed Internet connectivity. Thus, LEO has attracted considerable attention from both academia and industry in recent years [6,7,8,9].

To achieve ubiquitous and massive IoT connectivities, the access enabled by the LEO satellites will be the potential solution for supporting large-scale communications of future smart devices [2,10]. The LEO satellite communication (SatCom) system has the characteristics of global coverage (including the polar regions), on-demand access, and large capacity, which can effectively solve the shortage of the terrestrial communication system for IoT communications [11]. One promising use case for such consideration is that the LEO satellite can act as a base station to monitor disasters in the forest or mountainous area, such as fires, earthquakes, and mudslides, by collecting the sensing information on the IoT devices [12,13]. However, there are several challenges to the use of LEO satellites for IoT communications, notably in terms of spectrum shortage issues. On one hand, a growing variety of wireless communication systems lead to an increase in demand for radio spectrum. Nowadays, the available spectrum for satellites has already been allocated for specialized use such as remote sensing, navigation, telecommunications, etc. Thus, it is challenging to allocate a specified spectrum for LEO satellite-enabled IoT communications. On the other hand, unlike human-to-human communications, IoT communications are generally infrequent transmissions and occasionally exchange small amounts of data when transmitting [14,15,16]. In this regard, it would be wasteful to launch a dedicated satellite for IoT communications.

To deal with the above challenges, cognitive radio (CR) is a potential solution [17,18,19]. Specifically, CR allows the secondary users (SUs) to use the same spectrum as the primary users (PUs). In more detail, the PUs can use the spectrum resources without any constraint, while the SUs should use the same spectrum resources without significantly affecting the PU [20]. CR can be further categorized into overlay and underlay modes. In an overlay mode, SUs access the spectrum by sensing the PUs’ unused spectrums (spectrum holes), and when PUs appear, they must immediately release the occupied band. Therefore the overlay mode can minimize interference to PUs, while must take precise spectrum sensing for SUs, which consumes a lot of energy. This is very unfriendly to IoT devices, which are usually equipped with limited batteries and are very sensitive to energy consumption [21]. In an underlay mode, the SUs transmission can coexist with the PUs transmission simultaneously, provided that the caused interference to the primary transmission is below a tolerable threshold. In this way, IoT can transmit at any time without waiting for spectrum holes.

By introducing underlay CR into SatComs, IoT users can serve as the SUs and intelligently exploit any available LEO satellite spectrum while avoiding interference with legacy LEO satellite users [22,23]. In the cognitive LEO satellite system, it is important to prevent and minimize interference between the licensed PUs and SUs. This means that resource allocation and power control are inevitably applied to the secondary system to meet the constraints imposed by the interfering primary system [24,25,26,27]. Specifically, the spectrum sharing between the GEO and LEO SatCom systems is investigated in [28,29] to maximize the sum rate of the GEO and LEO SatCom systems and the sum rate of the LEO SatCom system, respectively. In [30], the power control algorithms are proposed to maximize the delay-limited capacity of the secondary system for a cognitive LEO satellite with terrestrial systems. In [31], the spectrum sharing in LEO and high-altitude platform (HAP) cognitive system is studied for resource allocation based on the imperfect spectrum sensing. The LEO satellite serves the sensors by sharing the spectrum of the GEO SatCom system [32]. The scenario where the satellite network reuses the spectrum resources of the terrestrial network is considered in [33] and the energy efficiency is maximized by designing the power control scheme. Nevertheless, most of the above works aim to optimize the resource of the secondary system under the fixed parameters for the primary transmission. Actually, the performance of the primary and secondary systems are coupled together. It is significant to propose a joint resource allocation scheme that is applicable to both legacy satellite users and cognitive satellite IoT users.

In this paper, we focus on an uplink cognitive LEO SatCom system to support IoT transmissions, in which the IoT communication system is served as the secondary system to share the spectrum of the legacy LEO SatCom system. In addition, the primary and secondary systems share the same receiver, i.e., the satellite base station. As the current LEO SatCom system widely uses code division multiple access (CDMA) technology [34,35,36,37], the spread spectrum signal is distributed over a wide frequency range, making it difficult to detect the power of the primary user range [38]. The collaborative underlay spectrum sharing model is more suitable for the cognitive LEO satellite IoT system than the overlay spectrum, which requires precise spectrum sensing. With perfect power control, the underlying IoT users can use the same spread spectrum as the legacy satellite users without degrading the PU’s communication quality, which greatly improves spectrum efficiency. In particular, IoT performance can be improved by using CDMA to successfully transmit more packets per unit time [39] and achieve better spectrum efficiency for IoT transmission than other orthogonal channel allocations [40]. Considering the properties of IoT transmission and in order to be compatible with the existing LEO system, in this paper, we consider supporting the cognitive satellite IoT communication in the CDMA manner. We are interested in the achievable rate and resource allocation in the cognitive LEO satellite networks. Specifically, the minimum mean square error (MMSE) detector is used to recover the information from the legacy LEO satellite users and the IoT users. Due to the randomness of spreading codes, it is difficult to allocate the resources directly. Thus, we use the random matrix theory to analyze the asymptotic signal-to-interference-plus-noise ratio (SINR) and obtain the achievable rate for both IoT and legacy systems. Moreover, we aim to jointly optimize the receive power of the legacy and IoT transmissions by maximizing the sum rate of the IoT users under the condition that the legacy satellite system can meet its performance requirement. To solve the formulated problem, we prove that the sum rate of the IoT users is quasi-concave over the legacy satellite user’s receive power. Based on such characteristics, we derive the optimal receive powers for these two systems. With the designed power allocation scheme, IoT users can achieve information transmission on the basis of ensuring the performance of the primary system. Finally, extensive simulation results are provided to validate the effectiveness of the designed power allocation scheme, which shows that IoT transmission can achieve information transmission on the basis of ensuring the performance of the primary system.

The rest of this paper is organized as follows. In Section 2, we build up the cognitive LEO satellite communication system model. In Section 3, we derive the asymptotic SINR for the legacy and IoT systems. In Section 4, the resource allocation problem is formulated and solved. Section 5 presents extensive simulation results which verify our theoretical analysis and validate the effectiveness of the proposed scheme. Finally, the paper is concluded in Section 7.

The notations used in this paper are listed as follows. The lowercase, boldface lowercase, and boldface uppercase letters *x*, x, and X denote a scalar variable (or constant), vector, and matrix, respectively. CN(μ,Σ) denotes the complex Gaussian distribution with mean μ and variance Σ. Notations $X^{T}$ and $X^{H}$ denote the transpose and conjugate transpose of matrix X, respectively. The notation $X^{*}$ denotes the optimal value of variable X. $I_{N}$ denotes the *N*-dimensional identity matrix. Notation E(·) denotes the statistical expectation. The notation A∘B denotes the Hadamard (element-wise) product.

## 2. System Model

**Figure 1:**
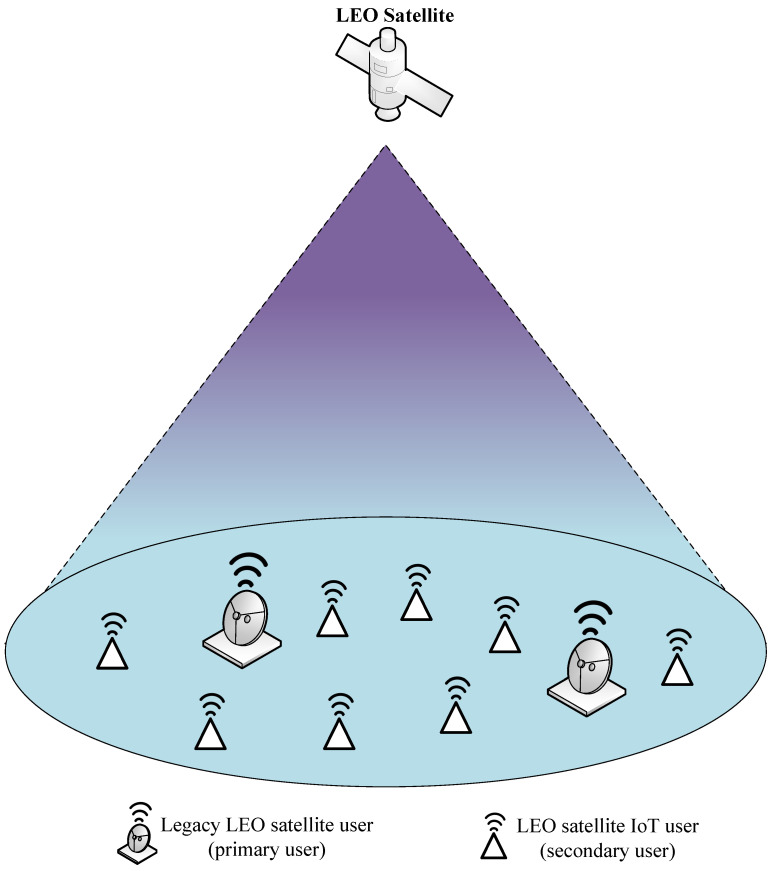
Cognitive LEO Satellite Communication System.

For the cognitive LEO satellite system, there are two types of networks: the legacy satellite network and the IoT network. The LEO satellite base station serves the legacy satellite users and the cognitive IoT users simultaneously in the CDMA manner. Meanwhile, IoT users share the same spectrum as legacy satellite users. The two kinds of systems also share the same LEO satellite receiving antenna. In this setup, the cross-channel state information (C-CSI) among the two systems is easy to obtain. We assume that the satellite and all user terminals are deployed with a single antenna. In the cognitive LEO satellite system, we consider one uplink case, as shown in Figure 1. Specifically, there are *U* primary users in the legacy satellite network and *K* secondary users in the IoT network. Each user in both the legacy satellite system and the IoT system is assigned a specific random spreading code with spreading gain *N*. In this way, the symbol duration of IoT is the same as that of the primary satellite.

In what follows, we will illustrate the channel model of the cognitive LEO satellite system and then the signal model followed by the achievable rates of both primary and secondary systems.

### 2.1. Channel Model

The satellite channel fading consists of two parts, including multipath propagation and shadow effect. The shadowed Rice model can effectively describe the two parts of fading effect, and has been widely applied in various frequency bands such as the UHF-band, L-band, S-band, and Ka-band channel analysis [41]. Specifically, the satellite channel fading coefficient between the satellite and the *m*-th user is given by

(1) $$\begin{array}{c} \begin{array}{c} {\tilde{h}}_{m}=A\exp\left(j\psi_{m}\right)+Z\exp\left(j\phi_{m}\right) \end{array} \end{array}$$

where $\psi_{m}\in \left[0,2\pi \right)$ is the stationary random phase and $\phi_{m}$ is the deterministic phase of the line-of-sight (LOS) component. *A* and *Z* are both independent stationery random processes. Specifically, *A* is the amplitude of the scatter component following Rayleigh distributions and *Z* is the amplitude of LOS component following the Nakagami distribution, which is given by

(2) $$\begin{array}{c} \begin{array}{cc} & p_{A}\left(a\right)=\frac{a}{b_{0}}\exp\left(\frac{-a^{2}}{2b_{0}}\right),a\geq 0\\ & p_{Z}\left(z\right)=\frac{2m^{m}}{\Gamma \left(m\right)\Omega^{m}}z^{2m-1}\exp\left(\frac{-mz^{2}}{\Omega }\right),z\geq 0 \end{array} \end{array}$$

Considering the atmospheric effects, the *m*-th user overall satellite channels can be expressed as

(3) $$\begin{array}{c} h_{m}=C_{L}{\tilde{h}}_{m} \end{array}$$

where $C_{L}=\lambda /\left(4\pi \sqrt{d_{0}^{2}+d_{m}^{2}}\right)$ denotes the free-space path loss coefficient with wavelength λ, the height of LEO satellite $d_{0}$, and the distance $d_{m}$ between the centers of the LEO satellite coverage area and *m*-th user.

Based on the above channel model, let $h_{u}$ denote the channel coefficient from the *u*-th legacy satellite user to the LEO satellite and $f_{k}$ denote the channel from the *k*-th IoT device to the LEO satellite. All of the channels $h_{u}$ and $f_{k}$ for u=1,⋯,U and k=1,⋯,K satisfy the expression of (3).

### 2.2. Signal Model

Denote by $a_{u}$ and $b_{k}$ the data symbol that legacy satellite user *u* transmits and the data symbol that IoT user *k* transmits, respectively. Symbol $a_{u}$ is spread by the spreading code $s_{u}$, and symbol $b_{k}$ is spread by the spreading code $t_{k}$. The spreading codes $s_{u}={\left[s_{u,1},s_{u,2},\cdots ,s_{u,N}\right]}^{T}$ and $t_{k}={\left[t_{k,1},t_{k,2},\cdots ,t_{k,N}\right]}^{T}$ satisfy the power constraints, i.e., $E[∥s_{u}{∥}^{2}]=P_{u}$, $E[∥t_{k}{∥}^{2}]={\tilde{P}}_{k}$ with the covariance of $E\left[s_{u}s_{u}^{H}\right]=\frac{P_{u}}{N}I_{N}$, $E\left[t_{k}t_{k}^{H}\right]=\frac{{\tilde{P}}_{k}}{N}I_{N}$, where $P_{u}$ and ${\tilde{P}}_{k}$ are the maximum transmit power of the *u*-th legacy satellite user and the *k*-th IoT device, respectively.

The total received signal at the satellite receiver corresponding to the *n*-th spreading code can be written as,

(4) $$\begin{array}{c} y_{n}=\sum_{u=1}^{U}h_{u}s_{u,n}a_{u}+\sum_{k=1}^{K}f_{k}t_{k,n}b_{k}+z_{n}, \end{array}$$

for n=1,⋯,N, where $z_{n}$ is the additive white Gaussian noise with $z_{n}∼CN\left(0,\sigma^{2}\right)$. For simplification, the received signal (4) can be rewritten as

(5) $$\begin{array}{c} y=H_{1}h\circ c_{1}+H_{2}f\circ c_{2}+z, \end{array}$$

where $H_{1}=\left[s_{1},s_{2},\cdots ,s_{U}\right]$ is N×U matrix formulated by the spreading codes of legacy satellite users, and $H_{2}=\left[t_{1},t_{2},\cdots ,t_{K}\right]$ is N×K matrix formulated by the spreading codes of IoT users, $h={\left[h_{1},h_{2},\cdots ,h_{U}\right]}^{T}$ is U×1 vector whose entries are the channel response of the legacy satellite users, $f={\left[f_{1},f_{2},\cdots ,f_{K}\right]}^{T}$ is K×1 vector composed by the channel resonses of the IoT users, $c_{1}={\left[a_{1},a_{2},\cdots ,a_{U}\right]}^{T}$ is U×1 vector whose entries are the data symbols the legacy satellite users transmit, $c_{2}={\left[b_{1},b_{2},\cdots ,b_{K}\right]}^{T}$ is K×1 vector composed by the data symbols the IoT users transmit, $z={\left[z_{1},z_{2},\cdots ,z_{N}\right]}^{T}$ is N×1 additive white Gaussian noise vector, i.e., $z∼CN(0,\sigma^{2}I_{N})$.

### 2.3. Achievable Rates

Suppose the MMSE detector is adopted by the LEO satellite to recover the information from both primary and secondary transmissions. For the primary transmission, let $w_{u}$ be the MMSE vector used to detect the *u*-th element of $c_{1}$, we can get

(6) $$\begin{array}{c} w_{u}^{H}y=w_{u}^{H}s_{u}h_{u}a_{u}+w_{u}^{H}\left(\sum_{i=1,i\neq u}^{U}s_{i}h_{i}a_{i}+\sum_{k=1}^{K}f_{k}b_{k}t_{k}+z\right). \end{array}$$

Thus, the SINR of the legacy satellite user *u* can be calculated as

$$\begin{array}{c} \gamma_{u,p}\;=\;\frac{|w_{u}^{H}s_{u}h_{u}{|}^{2}}{w_{u}^{H}(\sum_{i=1,i\neq u}^{U}|h_{i}{|}^{2}s_{i}s_{i}^{H}+\sum_{k=1}^{K}|f_{k}{|}^{2}t_{k}t_{k}^{H}+\sigma^{2}I_{N})w_{u}}. \end{array}$$

The MMSE vector $w_{u}$ is designed by minimizing the mean-square error (MSE) between the processed signal and the transmitted symbols. Define the MSE function for the legacy satellite user *u* as

(7) $$\begin{array}{c} J\left(w_{u}\right)=E[|a_{u}-w_{u}^{H}y{|}^{2}]. \end{array}$$

Then the MMSE vector $w_{u}$, which minimizes the MSE function $J(w_{u})$, is represented as

(8) $$\begin{array}{c} w_{u}=(\sum_{i=1}^{U}|h_{i}{|}^{2}s_{i}s_{i}^{H}+\sum_{k=1}^{K}|f_{k}{{|}^{2}t_{k}t_{k}^{H}+\sigma^{2}I_{N})}^{-1}s_{u}h_{u}. \end{array}$$

Based on the analysis in [42], the SINR of legacy satellite user *u* can be evaluated as

(9) $$\begin{array}{c} \gamma_{u,p}\;=\;|h_{u}{|}^{2}s_{u}^{H}(\sum_{i=1,i\neq u}^{U}|h_{i}{|}^{2}s_{i}s_{i}^{H}+\sum_{k=1}^{K}|f_{k}{{|}^{2}t_{k}t_{k}^{H}+\sigma^{2}I_{N})}^{-1}s_{u}. \end{array}$$

Accordingly, the achievable rate for the *u*-th legacy satellite user can be written as

(10) $$\begin{array}{c} R_{u,p}=\frac{1}{N}\log\left(1+\gamma_{u,p}\right). \end{array}$$

Similarly, we use the MMER detector to recover the transmitted signal by the IoT users. Based on the above analysis, we can obtain the SINR of the IoT users. Specifically, the SINR of the *k*-th IoT user is given by

(11) $$\begin{array}{c} \gamma_{k,s}\;=\;|f_{k}{|}^{2}t_{k}^{H}(\sum_{i=1,i\neq k}^{K}|f_{i}{|}^{2}t_{i}t_{i}^{H}+\sum_{u=1}^{U}|h_{u}{{|}^{2}s_{u}s_{u}^{H}+\sigma^{2}I_{N})}^{-1}t_{k}. \end{array}$$

Then, the achievable rate for the *k*-th IoT user can be written as

(12) $$\begin{array}{c} R_{k,s}=\frac{1}{N}\log\left(1+\gamma_{k,s}\right). \end{array}$$

From (9) and (11), we can find that the legacy satellite user’s SINR depends on the spreading codes of the primary system as well as those of the secondary system. Because of the randomness of spreading codes, it is difficult to exactly calculate the SINR of the legacy satellite user and allocate resources for these two systems. Thus, in Section 3, we will analyze the asymptotic SINRs for both primary and secondary transmissions.

## 3. Asymptotic Analysis

In this section, we analyze the asymptotic SINRs for both primary and secondary systems in order to allocate the resource for the two systems.

We consider a large cognitive LEO SatCom system, in which the number of users is large, i.e., U→∞ and K→∞. To support a large number of users, it is reasonable to scale up *N* as well, i.e., N→∞, but U/N converges to a constant parameter $\alpha_{1}$, which represents the legacy satellite system load. Similarly, we have that K/N converges to a constant parameter $\alpha_{2}$, which represents the IoT system load.

To analyze the asymptotic SINR, we first present the following proposition.

**Proposition** **1** (Theorem 3.1 of [42]). *For a symbol-synchronous multi-access spread-spectrum system with spreading gain N, the SINR $\gamma_{1}$ for the *1*-st user in a M user system is deterministic and approximately satisfies,*

(13) $$\gamma_{1}=\frac{p_{1}}{\sigma^{2}+\frac{1}{N}\sum_{i=2}^{M}I\left(p_{i},p_{1},\gamma_{1}\right)},$$

*where*

(14) $$I\left(p_{i},p_{1},\gamma_{1}\right)=\frac{p_{i}p_{1}}{p_{1}+p_{i}\gamma_{1}},$$

*and $p_{i}$ denotes the received power of the user i.*

From Proposition 1, the asymptotic SINR is determined by the received power for each user. Based on (13) and (14), the asymptotic SINR of the *u*-th legacy satellite user satisfies:

(15) $$\begin{array}{c} \gamma_{u,p}=\frac{P_{u}{\left|h_{u}\right|}^{2}}{\frac{1}{N}\left(\sum_{i\;=\;1,i\neq u}^{U}\frac{P_{u}|h_{u}{|}^{2}P_{i}{\left|h_{i}\right|}^{2}}{P_{u}|h_{u}{|}^{2}+P_{i}{\left|h_{i}\right|}^{2}\;\gamma_{u,p}}+\sum_{i=1}^{K}\frac{P_{u}|h_{u}{|}^{2}{\tilde{P}}_{i}{\left|f_{i}\right|}^{2}}{P_{u}|h_{u}{|}^{2}+{\tilde{P}}_{i}{\left|f_{i}\right|}^{2}\gamma_{u,p}}\;\right)+\sigma^{2}}, \end{array}$$

As both the primary and secondary systems are based on CDMA, the method for analyzing the asymptotic SINR for the IoT users is the same as the one for the legacy satellite system. Thus, the asymptotic SINR of the *k*-th IoT user is given by:

(16) $$\begin{array}{cc} \; & \gamma_{k,s}=\frac{{\tilde{P}}_{k}{\left|f_{k}\right|}^{2}}{\frac{1}{N}\left(\sum_{i=1,i\neq k}^{K}\frac{{\tilde{P}}_{k}|f_{k}{|}^{2}{\tilde{P}}_{i}{\left|f_{i}\right|}^{2}}{{\tilde{P}}_{k}|f_{k}{|}^{2}+{\tilde{P}}_{i}{\left|f_{i}\right|}^{2}\gamma_{k,s}}+\sum_{i=1}^{U}\frac{{\tilde{P}}_{k}|f_{k}{|}^{2}P_{i}{\left|h_{i}\right|}^{2}}{{\tilde{P}}_{k}|f_{k}{|}^{2}+P_{i}{\left|h_{i}\right|}^{2}\gamma_{k,s}}\right)+\sigma^{2}}, \end{array}$$

From (15) and (16), we find that the asymptotic SINRs for both primary and secondary systems are related to the received power for all of the links. To simplify the process of resource allocation, we assume that the received powers for legacy satellite users are the same one, which is given by *q*. This is achieved by perfectly power control. Similarly, the received power for the IoT users is the same one, which is given by *p*. Then, (15) can be simplified as

(17) $$\gamma_{u,p}=\frac{q}{\sigma^{2}+\frac{U}{N}\frac{q}{1+\gamma_{u,p}}+\frac{K}{N}\frac{pq}{p+q\gamma_{u,p}}},$$

which gives

(18) $$\begin{array}{c} \gamma_{p}=\frac{q}{\frac{\alpha_{1}q}{1+\gamma_{p}}+\frac{\alpha_{2}pq}{q+p\gamma_{p}}+\sigma^{2}}. \end{array}$$

Similarly, (16) can be simplified as

(19) $$\begin{array}{c} \gamma_{s}=\frac{p}{\frac{\alpha_{2}p}{1+\gamma_{s}}+\frac{\alpha_{1}pq}{p+q\gamma_{s}}+\sigma^{2}}. \end{array}$$

## 4. Joint Resource Allocation in Coginitve LEO SatComm System

In this section, we will formulate and solve the resource allocation schemes to maximize the sum rate of all IoT users under some constraints. First, we will first investigate the optimal IoT receive power and the optimal joint legacy satellite use and IoT user receive powers. Then, based on the solved optimal power, we will exploit the optimal number of IoT users. Finally, we will discuss the effect of the non-synchronous between the primary and secondary systems.

### 4.1. Resource Allocation

To protect the legacy satellite service, we have to guarantee its SINR no less than the target value, $\beta^{*}$, i.e., $\gamma_{p}\geq \beta^{*}$, and guarantee the IoT user receive power no more than the limit value, $\bar{P}$, i.e., $p\leq \bar{P}$. Based on the above analysis, our first resource allocation problem in the CR system tries to maximize the sum rate for the IoT system, which can be formulated as P1,

$$\begin{array}{cc} \max_{p} & \;\;\;\;\frac{K}{N}\log\left(1+\gamma_{s}\right)\\ s.t. & \;\;\;\;\gamma_{p}\geq \beta^{*}\\ \; & \;\;\;\;p\leq \bar{P}. \end{array}$$

From (19), it is difficult to get the closed-form expression for $\gamma_{s}$. Similarly, the closed-form expression for $\gamma_{p}$ is also difficult to obtain. To overcome this problem, we provide the following lemma.

**Lemma** **1.** *For any $\alpha_{2}$ and q, if we have $p_{1}>p_{2}$, then, we have $\gamma_{1,s}>\gamma_{2,s}$.*

**Proof**. Please see Appendix A.    □

From Lemma 1, we can find that the objective function increases with the growth of *p*. However, the increase in *p* will decrease the asymptotic SINR of the legacy satellite users. Similar to the Lemma 1, if $q_{1}>q_{2}$, then, $\gamma_{1,p}>\gamma_{2,p}$. Thus, when *q* is small, the SINR requirement constraint, i.e., $\gamma_{p}\geq \beta^{*}$ is dominant while the power limit is not effective. So ignoring the power constraint, we can get P2:

(20) $$\begin{array}{c} \begin{array}{cc} \max_{p}\; & \frac{K}{N}\log\left(1+\gamma_{s}\right)\\ s.t.\; & \gamma_{p}\geq \beta^{*}. \end{array} \end{array}$$

For the optimization problem P2, when *p* is at the maximum value, the sum rate of the IoT users will be at the maximum value, while $\gamma_{p}$ will decrease. Thus, the objective function is maximized when the equality constraint holds, i.e., $\gamma_{p}=\beta^{*}$. Accordingly, we can derive the optimal *p* of the IoT receive power as follows

(21) $$p=\frac{\frac{q}{\beta^{*}}-\sigma^{2}-\frac{\alpha_{1}q}{1+\beta^{*}}}{\alpha_{2}+\frac{\sigma^{2}\beta^{*}}{q}+\frac{\alpha_{1}\beta^{*}}{1+\beta^{*}}-1}.$$

When *q* is large enough, the value of *p* has achieved the power constraint while $\gamma_{s}$ does not reach $\beta^{*}$. So ignoring the first constraint, we can get P3:

(22) $$\begin{array}{c} \begin{array}{cc} \max_{p}\; & \frac{K}{N}\log\left(1+\gamma_{s}\right)\\ s.t.\; & p\leq \bar{P}. \end{array} \end{array}$$

Due to the fact that $\gamma_{s}$ will increase with *p*, the optimal receive power for the IoT users is

(23) $$p=\bar{P}.$$

### 4.2. Joint Resource Allocation

From (21), we know that the optimal receive power of the IoT users is relevant to the received power of the legacy satellite system. With perfect power control, we could adjust the receive power *q* of the legacy satellite users to maximize the sum rate of the IoT users. Thus, we formulate the joint resource allocation problem as P4:

$$\begin{array}{cc} \max_{p,q} & \;\;\;\;\frac{K}{N}\log\left(1+\gamma_{s}\right)\\ s.t. & \;\;\;\;\gamma_{p}\geq \beta^{*}\\ \; & \;\;\;\;p\leq \bar{P}. \end{array}$$

For every given *q*, there must be an optimal IoT user receive power *p* which can ensure the maximization of IoT system’s sum rate. To overcome the joint resource allocation problem, we will find the influence of *q* to $\gamma_{s}$ with the optimal IoT user receive power *p*, based on which we provide the following analysis.

**Lemma** **2.** *When $q\in [q_{0},q_{1})$ with the optimal IoT user receive power p, if $q_{1}>q_{2}$, then, we can have $\gamma_{1,s}>\gamma_{2,s}$.*

**Proof**. The proof is given in Appendix B.    □

**Lemma** **3.** *When $q\in [q_{1},\infty )$, with the optimal IoT user receive power p, if $q_{1}>q_{2}$, then, we can have $\gamma_{1,s}<\gamma_{2,s}$.*

**Proof**. The proof is given in Appendix C.    □

Note that the values of $q_{0}$ and $q_{1}$ will be discussed later. From Lemmas 2 and 3, we know that when $q\in [q_{0},q_{1})$, $\gamma_{s}$ increases with *q*, and when $q\in [q_{1},\infty )$, $\gamma_{s}$ decreases with *q*. This indicates that the sum rate of the IoT systems is quasi-concave over the legacy satellite receive power. As a result, when $q=q_{1}$, $p=\bar{P}$, the sum rate of the IoT system is maximized. Meanwhile, if we have $q_{0}>q_{1}$, the IoT users can not transmit signals i.e., p=0.

Next, we will focus on the investigation of the values of $q_{0}$ and $q_{1}$. When the legacy satellite system cannot tolerate the interference of the IoT system, the receive power of legacy satellite system is minimal, i.e.,

(24) $$\begin{array}{c} \beta^{*}=\frac{q_{0}}{\sigma^{2}+\frac{q_{0}\alpha_{1}}{1+\beta^{*}}}. \end{array}$$

Solving this equation for $q_{0}$, we can get

(25) $$q_{0}=\frac{\sigma^{2}}{\frac{1}{\beta^{*}}-\frac{\alpha_{1}}{1+\beta^{*}}}.$$

When $q=q_{1}$, (21) and (23) both hold, which gives

(26) $$\bar{P}=\frac{\frac{q_{1}}{\beta^{*}}-\sigma^{2}-\frac{\alpha_{1}q_{1}}{1+\beta^{*}}}{\alpha_{2}+\frac{\sigma^{2}\beta^{*}}{q_{1}}+\frac{\alpha_{1}\beta^{*}}{1+\beta^{*}}-1}.$$

By solving (26), we can get the result of $q_{1}$.

As aforementioned, the optimal joint resource allocation scheme is related to the primary and secondary system loads, i.e., $\alpha_{1}$ and $\alpha_{2}$. Intuitively, the growth of the number of IoT users will contribute to the new increments in the sum rate, however, leading to the increase in interference. Thus, there exists a tradeoff in the number of IoT users. In the following, we aim to exploit the optimal user number of the IoT system to maximize the sum rate of the IoT system for a given *N*.

### 4.3. Optimal IoT User Number

Here, we will illustrate the optimal IoT user number to maximize the sum rate of the IoT transmissions, subject to the receive power constraint and the primary SINR requirement constraint, which can be mathematically formulated as P5:

(27) $$\begin{array}{c} \begin{array}{ccc} \max_{p,q,K} & & \frac{K}{N}\log\left(1+\gamma_{s}\right)\\ s.t. & & \left\{\begin{array}{cc} & \gamma_{p}\geq \beta^{*}\\ & p\leq \bar{P}. \end{array}\right. \end{array} \end{array}$$

For every given *K*, there exists one optimal *p* and *q*, which can ensure the maximization of the sum rate of the IoT system. When given *p* and *q*, the optimization of *K* involves the log function and non-closed formula of $\gamma_{s}$. Thus, it is difficult to gain the closed-form of the optimal *K* when given *p* and *q*. In fact, when *K* is small, the sum rate of the IoT users increases with the growth of *K*. When *K* is large, the sum rate of the IoT users decreases with the growth of *K* since in this case, the SINR of the IoT users will dominate the sum rate of the IoT system. Based on this fact, we can apply the one-dimensional search scheme to find the optimal tuple $\left(p^{*},q^{*},K^{*}\right)$ to solve the optimization problem P5. Specifically, for each *K*, we calculate the optimal $p^{*}$ and $q^{*}$. By searching different *K*, we can gain multiple tuples $\left(p^{*},q^{*},K\right)$. By comparing the sum rate of the IoT system with $\left(p^{*},q^{*},K\right)$, we can obtain the optimal tuple $\left(p^{*},q^{*},K^{*}\right)$. The details of the proposed algorithm for solving problem P5 are summarized in Algorithm 1. Specifically, we first initialize *K* and ζ, where the initial *K* is a small number and ζ is greater than one. Then, we iteratively update *p*, *q*, and *K* until the objective function gradually decreases.
**Algorithm 1.** Solution to P5Initialize $K^{\left(0\right)},\zeta$ and set t=0;**Repeat**t←t+1Calculate $q^{\left(t\right)}$ and $p^{\left(t\right)}$ based on (26) given $K^{\left(t-1\right)}$;$K^{\left(t\right)}=\zeta K^{\left(t-1\right)}$;**Until** the objective function of P5 gradually decreases.Obtain optimal tuple $\left(p^{*},q^{*},K^{*}\right)$.

### 4.4. Non-Synchronous Uplink

In the above, the entire analysis is that of a synchronous uplink cognitive LEO satellite spectrum sharing system. In this section, we extend to the non-synchronous case.

For the non-synchronous uplink cognitive LEO SatCom system, the total received signal at the satellite base station can be written as,

(28) $$\begin{array}{c} y=H_{1}h\circ c_{1}+H_{3}f\circ c_{3}+z, \end{array}$$

where $H_{3}=\left[t_{L},t_{L+1},\cdots ,t_{K},t_{1},t_{2},\cdots ,t_{L-1}\right]$ is N×K matrix formulated by the spreading codes of the IoT users, $c_{3}={\left[b_{L},b_{L+1},\cdots ,b_{K},b_{1},b_{2},\cdots ,b_{L}\right]}^{T}$ is K×1 vector composed by the data symbols the IoT users transmit, and *L* is the synchronous error between the primary and the secondary systems. Based on the discussions in Section 2, we can obtain the MMSE output for the non-synchronous uplink cognitive LEO SatCom system, which is given by

(29) $$\begin{array}{c} w_{u}^{H}y=w_{u}^{H}s_{u}a_{u}+w_{u}^{H}\left(\sum_{i=1,i\neq u}^{U}s_{i}a_{i}+\sum_{k=1}^{K}b_{k}t_{k}+n\right). \end{array}$$

From (29), we can find that although the primary and secondary systems are not synchronous perfectly, the MMSE output of the non-synchronous uplink system is similar to that of the synchronous uplink system. Thus, the analysis and scheme of resource allocation of the non-synchronous uplink system are similar to that of the synchronous uplink system and thereby omitted.

## 5. Simulations Results

In this section, simulation results are presented to evaluate the performance of the proposed cognitive LEO satellite communication system. The spreading gain for the legacy and IoT systems is set to N=256, which is large enough to verify the asymptotic results obtained in this paper. Although the LEO satellite system involves the shadowed Rice channel, the resource allocation schemes in this paper focus on the receive signal-to-noise ratio (SNR). Due to the fact that the performance of the legacy and IoT systems is related to the SINR, here, we use the receive SNR, i.e., $\frac{p}{\sigma^{2}}$ and $\frac{q}{\sigma^{2}}$, to evaluate the performance of the proposed cognitive LEO satellite communication system. Specifically, the white Gaussian noise power is normalized to $\sigma^{2}=1$. We set the target SINR threshold $\beta^{*}$ for the legacy satellite system to 5 dB. To show the effectiveness of our proposed framework and algorithms, we set two benchmarks, which are illustrated as follows:

- **Benchmark 1**: To show the advantages of spectrum sharing, we show the performance of the legacy satellite systems without spectrum sharing, whose SINR with the MMSE detector is given by $\gamma_{l}=\frac{q}{\frac{\alpha_{1}q}{1+\gamma_{l}}+\sigma^{2}}$. Accordingly, the sum rate of the legacy satellite users is given by $\frac{U}{N}\log\left(1+\gamma_{l}\right)$.
- **Benchmark 2**: To show the advantages of the joint resource allocation scheme, we show the performance by only optimizing the receive power of the IoT devices based on the analysis in Section 4.1.

**Figure 2:**
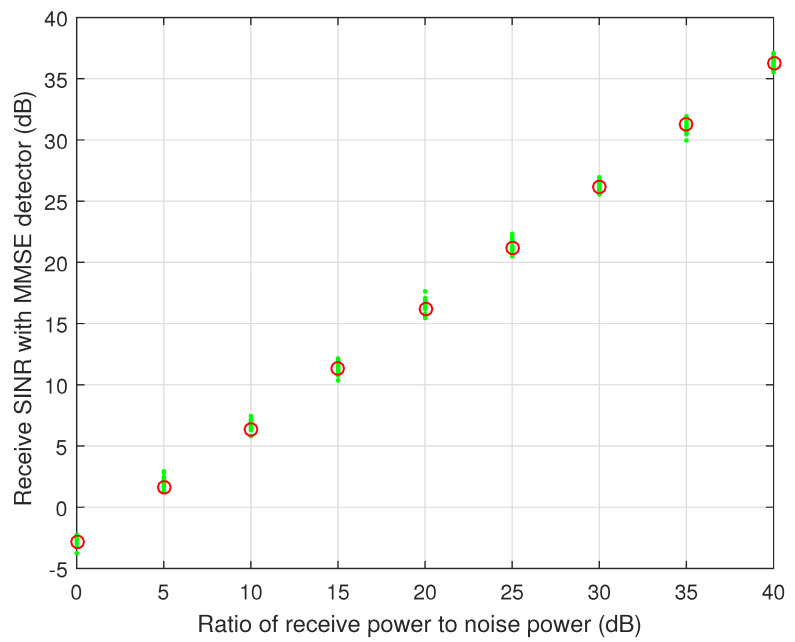
Received SINR with MMSE detector vs. received SNR, i.e., $\frac{q}{\sigma^{2}}$, for the simulated and theoretical results.

Firstly, we evaluate the asymptotic SINR of the legacy satellite system by comparing the simulated SINR with the theoretical SINR with the MMSE detector. We set U=50, K=100, and p=20 dB. The calculation of simulated SINR is based on random spreading codes which are assigned to each user in the legacy satellite system, as shown in (9). The theoretical SINR with the MMSE detector can be calculated by (18). As shown in Figure 2, the dense small circles are the simulated SINRs, while the big circles are the theoretical SINRs for the MMSE detector. It is seen that the theoretical SINR can be considered as the statistical mean value of the simulated SINRs. Thus, it is reasonable to formulate the joint resource allocation problem based on the asymptotic SINR. In addition, we can find that the interference from the IoT users and other legacy satellite users leads to about 4 dB SINR loss with MMSE detector compared with the SNR of the effective legacy satellite user.

**Figure 3:**
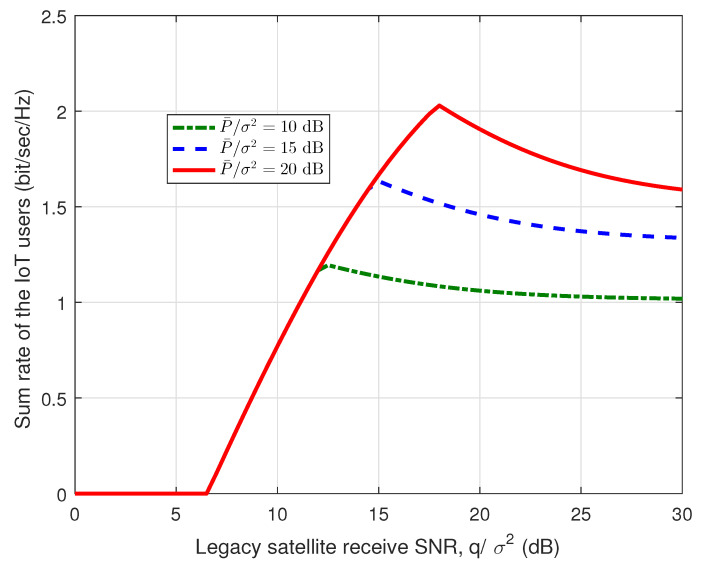
Sum rate of the IoT users vs. legacy satellite receive SNR.

**Figure 4:**
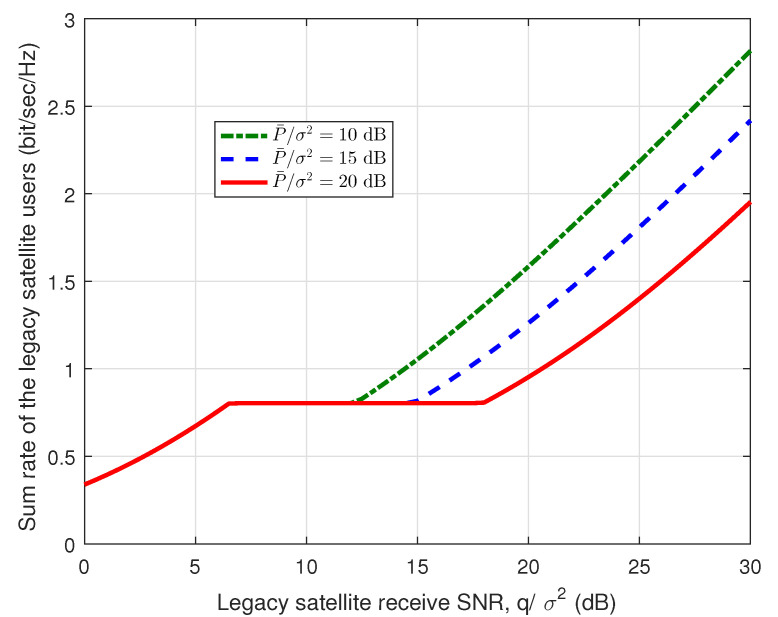
Sum rate of the legacy satellite users vs. legacy satellite receive SNR.

Figure 3 and Figure 4 present the sum rate of the IoT users and the sum rate of the legacy satellite users w.r.t. the legacy satellite receive SNR, i.e., $\frac{q}{\sigma^{2}}$, respectively. Here, we set U=100 and K=200. From Figure 3, it can be found that the sum rate of the IoT users is quasi-concave over the legacy satellite receive SNR, which is consistent with the results in Lemmas 2 and 3. In addition, from Figure 3 and Figure 4, we can find that when $\frac{q}{\sigma^{2}}<6.5$ dB, the sum rate of the IoT users is equal to 0, while the sum rate of the legacy satellite users increases with the growth of $\frac{q}{\sigma^{2}}$. The main reason is that when $\frac{q}{\sigma^{2}}<6.5$ dB, we have $\frac{q}{\sigma^{2}}<\frac{q_{0}}{\sigma^{2}}$, which means that the legacy satellite system cannot tolerate the interference of the IoT system. For $\frac{p}{\sigma^{2}}=10$ dB, the sum rate of the IoT users increases with $\frac{q}{\sigma^{2}}$, when $\frac{q}{\sigma^{2}}<12.5$ dB. In this case, the sum rate of the legacy satellite users remains unchanged, which indicates that the legacy SINR requirement constraint dominates the power allocation scheme. When the IoT receive power limit is not achieved, for each value of $\frac{p}{\sigma^{2}}$, the curves are coincident and all the interference margins can be exploited by the IoT system. Note that the interference margin refers to the tolerable interference of the legacy satellite system. When $\frac{q}{\sigma^{2}}<12.5$ dB, the receive power limit dominates the power allocation scheme. Note that the tuning points in the two figures are related to the parameters design. With other parameters, the tuning points may change.

**Figure 5:**
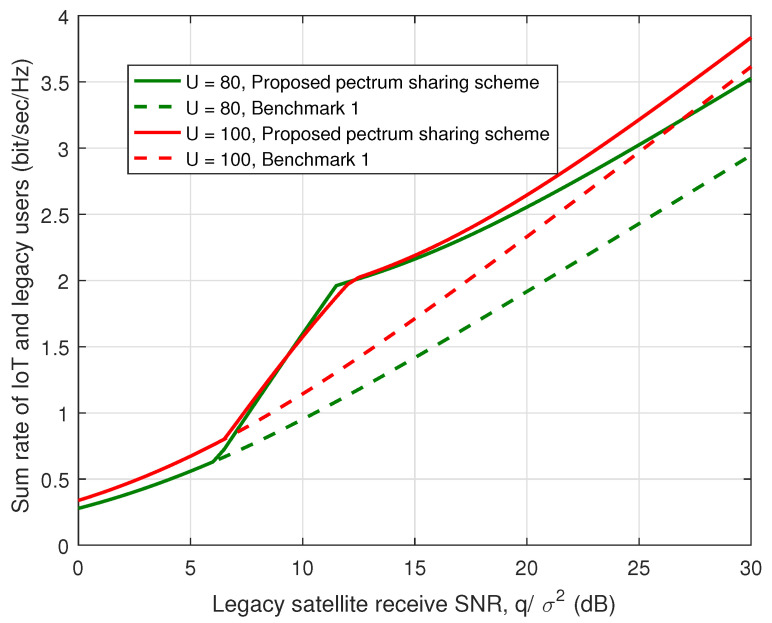
Sum rate of IoT and legacy satellite users vs. legacy satellite receive SNR.

Figure 5 presents the sum rate of the IoT and the legacy satellite users w.r.t. the legacy satellite receive SNR, i.e., $\frac{q}{\sigma^{2}}$ for different legacy user numbers with $\frac{\bar{P}}{\sigma^{2}}=10$ dB. As aforementioned, Benchmark 1 indicates the performance of the legacy satellite users without spectrum sharing. From Figure 5, it is obvious that the proposed spectrum sharing scheme has a higher sum rate, which indicates the effectiveness of the spectrum sharing scheme. Meanwhile, from this figure, we can find that the spectrum sharing scheme has greater performance gains when the number of legacy satellite users is small. The main reason is that the available interference margin increases with the decrease of the legacy user number. In addition, when $\frac{q}{\sigma^{2}}<6.5$ dB, the two schemes have the same performance since the legacy satellite system cannot tolerate the interference of the IoT system and the sum rate of the IoT users is equal to 0.

**Figure 6:**
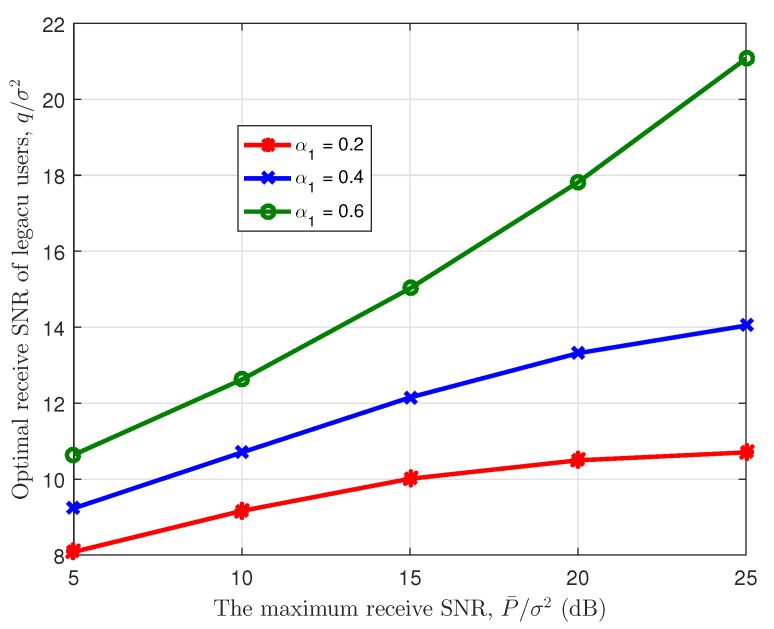
Optimal legacy satellite receive SNR vs. maximum IoT receive SNR.

**Figure 7:**
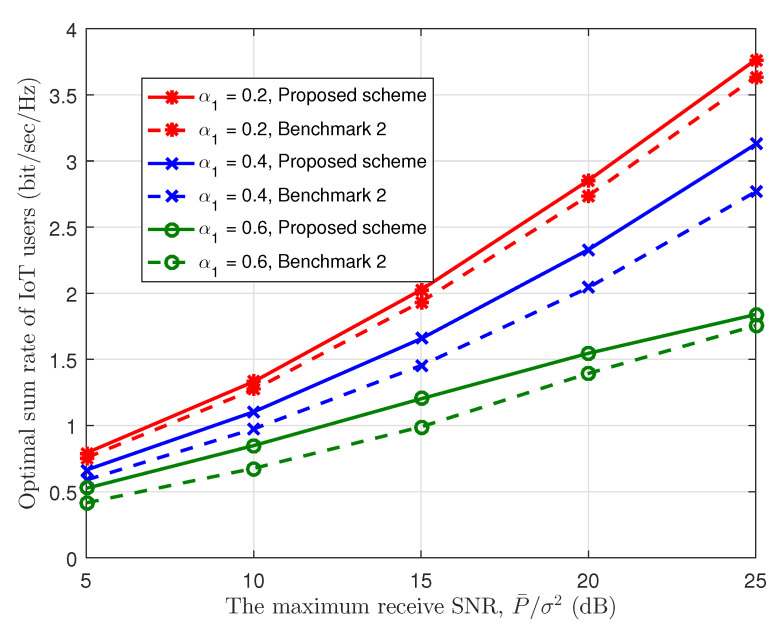
Optimal sum rate of IoT users vs. maximum IoT receive SNR.

Next, we evaluate the optimal legacy satellite receive power and the optimal sum rate of the IoT user based on the optimized joint power allocation scheme by varying the maximum receive SNR of the IoT users $\frac{\bar{P}}{\sigma^{2}}$, as shown in Figure 6 and Figure 7. Meanwhile, to show the effectiveness of the joint resource allocation scheme, we also plot the curves of Benchmark 2 in Figure 7, where we set the $\frac{q}{\sigma^{2}}=20$ dB. In the two figures, we set K=150. From Figure 6, we can find that when $\frac{\bar{P}}{\sigma^{2}}$ grows, the optimal receive SNR of the legacy user, i.e., $\frac{q_{1}}{\sigma^{2}}$, also increases. This indicates that the sum rate of the IoT users is maximized with higher $\frac{q_{1}}{\sigma^{2}}$, which is also shown in Figure 3. Meanwhile, the optimal receive SNR increase with $\alpha_{1}$ due to less interference from the legacy satellite users. In Figure 7, we can observe that, for any $\alpha_{1}$, as $\frac{\bar{P}}{\sigma^{2}}$ increases, the optimal IoT sum rate increases since the higher $\frac{\bar{P}}{\sigma^{2}}$ can help the IoT users to exploit more interference margin. Furthermore, we can find our proposed joint resource allocation scheme performs better than Benchmark 2 for any legacy system load, which shows the effectiveness of the joint design. In addition, the optimal IoT sum rate decreases with $\alpha_{1}$ since the available interference margin decreases with the increase of $\alpha_{1}$.

**Figure 8:**
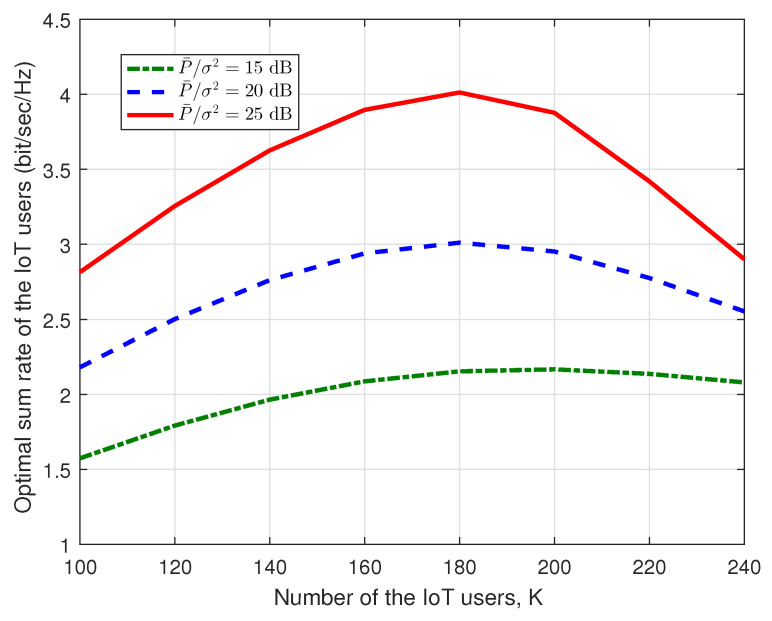
Optimal sum rate of the IoT users vs. the number of the IoT users.

Finally, the optimal sum rate of the IoT users w.r.t. the number of the IoT users is shown in Figure 8. In this figure, we set U=50. For every given *K*, the calculation of the sum rate of the IoT users is based on the optimal *p* and *q* which can ensure the maximization of the IoT sum rate. It is observed that the optimal sum rate of the IoT users is quasi-concave over the number of the IoT users since the sum rate of the IoT system equals the user number times each user’s rate, and when the user number increases, this, however, decreases the SINR in (19), resulting in the decrease of each user’s throughput. We also know that the user number must be an integer. Thus, we can traverse *K* next to the peak in Figure 8 to confirm the user number of the IoT system which can make the IoT sum rate reaches the maximum. For example, from Figure 8, we can observe that when $\frac{\bar{P}}{\sigma^{2}}=25$ dB, the optimal secondary user number is around 180. Meanwhile, we can find that in this setup, the receive SNR $\frac{\bar{P}}{\sigma^{2}}$ has a trivial effect on the optimal number of the IoT users due to the huge effect of the QoS requirements of the legacy satellite users.

## 6. Discussions

Due to the difficulty in launching a dedicated satellite and allocating a specified spectrum for IoT communications, we propose one framework, which uses the legacy satellite and its authorized spectrum to support the IoT transmissions. Due to the interference between the legacy satellite users and the IoT users, we propose one joint resource allocation scheme to balance the two types of transmissions, where the legacy satellite users have higher priority. Simulation results show the advantages and the effectiveness of the spectrum sharing and joint resource allocation scheme. In a nutshell, the main contributions of this paper are summarized as follows.

- The cognitive LEO SatCom system is proposed to support IoT transmissions, which can effectively enhance the coverage and spectrum efficiency.
- Due to the randomness of spreading codes, we use the random matrix theory to analyze the asymptotic SINR and obtain the achievable rate for IoT and legacy systems.
- The receive power of the legacy and IoT transmissions are jointly optimized and the closed-form expressions of the optimal receive powers for these two systems are derived.
- Extensive simulation results are provided to validate the effectiveness of the designed power allocation scheme, which shows that IoT devices can achieve information transmission on the basis of ensuring the performance of the primary system.

## 7. Conclusions

In this paper, we have proposed an uplink cognitive LEO SatCom system to enable IoT communication by sharing the spectrum of the legacy satellite system. Specifically, the legacy satellite users and the IoT users are served by the same LEO satellite in the CDMA manner. Considering the randomness of spreading codes, we have analyzed the asymptotic SINRs by using the random matrix theory and obtained the achievable rates for both legacy and IoT systems. We have proposed joint resource allocation schemes for the uplink cognitive LEO SatCom system, which aims to maximize the sum rate of the IoT users, with the consideration to protect the legacy primary satellite users. It has been proved that the sum rate of the IoT users is quasi-concave over legacy satellite user receive power, based on which the optimal receive powers for these two systems are derived. Simulation results have verified our theoretical analysis and validated the effectiveness of our scheme to achieve the optimal sum rate of the IoT users in the cognitive LEO SatCom system.

## Data Availability

Data is unavailable due to privacy.

## Appendix A. Proof of Lemma 1

It is difficult to prove Lemma 1 directly, but we can use the proof by contradiction. Based on (19), we have

(A1) $$\frac{\gamma_{1,s}}{\gamma_{2,s}}=\frac{p_{1}}{p_{2}}\frac{\frac{\alpha_{2}p_{2}}{1+\gamma_{2,s}}+\frac{\alpha_{1}p_{2}q}{p_{2}+q\gamma_{2,s}}+\sigma^{2}}{\frac{\alpha_{2}p_{1}}{1+\gamma_{1,s}}+\frac{\alpha_{1}p_{1}q}{p_{1}+q\gamma_{1,s}}+\sigma^{2}}.$$

By simplifying the above, we have

(A2) $$\begin{array}{cc} \; & \sigma_{2}\left(p_{2}\gamma_{1,s}-p_{1}\gamma_{2,s}\right)+\alpha_{2}p_{1}p_{2}\left(\frac{1}{1+\gamma_{2,s}}-\frac{1}{1+\gamma_{1,s}}\right)\\ \; & \;\;+\alpha_{1}p_{1}p_{2}q\left(\frac{p_{2}\gamma_{1,s}-p_{1}\gamma_{2,s}}{\left(p_{1}+q\gamma_{1,s}\right)\left(p_{2}+q\gamma_{2,s}\right)}\right)=0. \end{array}$$

For $p_{1}>p_{2}$, if we assume $\gamma_{1,s}\leq \gamma_{2,s}$, then, we have

$$p_{2}\gamma_{1,s}-p_{1}\gamma_{2,s}<0,$$

$$\frac{1}{1+\gamma_{2,s}}-\frac{1}{1+\gamma_{1,s}}\leq 0,$$

$$\frac{p_{2}\gamma_{1,s}-p_{1}\gamma_{2,s}}{\left(p_{1}+q\gamma_{1,s}\right)\left(p_{2}+q\gamma_{2,s}\right)}<0,$$

which means that (A2) cannot be satisfied. Hence, for $p_{1}>p_{2}$, we have $\gamma_{1,s}>\gamma_{2,s}$. Thus, Lemma 1 is proved.

## Appendix B. Proof of Lemma 2

Simplifying (19), we have

(A3) $$\frac{\sigma^{2}\gamma_{s}}{p}-\frac{\alpha_{2}}{1+\gamma_{s}}-\frac{\alpha_{1}}{1+\frac{q}{p}\gamma_{s}}+\alpha_{1}+\alpha_{2}-1=0.$$

Substituting $p_{1}$, $p_{2}$, $q_{1}$, $q_{2}$, $\gamma_{1,s}$, and $\gamma_{2,s}$ into (A3) and subtracting the two equations yields

(A4) $$\begin{array}{cc} \; & \sigma^{2}\left(\frac{\gamma_{1,s}}{p_{1}}-\frac{\gamma_{2,s}}{p_{2}}\right)+\frac{\alpha_{2}}{1+\gamma_{2,s}}-\frac{\alpha_{2}}{1+\gamma_{1,s}}\\ \; & +\frac{\alpha_{1}}{1+\frac{q_{2}}{p_{2}}\gamma_{2,s}}-\frac{\alpha_{1}}{1+\frac{q_{1}}{p_{1}}\gamma_{1,s}}=0. \end{array}$$

For $q_{1}>q_{2}$, if we assume $\gamma_{1,s}\leq \gamma_{2,s}$, then, we have the following analysis.

It is obvious that

(A5) $$\frac{\alpha_{2}}{1+\gamma_{2,s}}-\frac{\alpha_{2}}{1+\gamma_{1,s}}\leq 0.$$

Based on (21), it is difficult to find out the relation between *p* and *q* directly. Thus, (21) is deformed as

(A6) $$\frac{1}{\beta^{*}}=\frac{\alpha_{1}}{1+\beta^{*}}+\frac{\alpha_{2}}{\frac{q}{p}+\beta^{*}}+\frac{\sigma^{2}}{q}.$$

From (A6), it is obvious that if *p* decreases with *q*, then Equation (A6) is impossible to be satisfied. That is to say, if $q_{1}>q_{2}$, then, we have $p_{1}>p_{2}$. Thus,

(A7) $$\frac{\gamma_{1,s}}{p_{1}}-\frac{\gamma_{2,s}}{p_{2}}<0.$$

Since

$$\frac{q}{p}=\frac{\alpha_{2}+\frac{\sigma^{2}\beta^{*}}{q}+\frac{\alpha_{1}\beta^{*}}{1+\beta^{*}}-1}{\frac{1}{\beta^{*}}-\frac{\sigma^{2}}{q}-\frac{\alpha_{1}}{1+\beta^{*}}},$$

it is easy to see that q/p decreases with *q*, i.e., $q_{2}/p_{2}>q_{1}/p_{1}$. Thus,

$$\frac{q_{2}}{p_{2}}\gamma_{2,s}>\frac{q_{1}}{p_{1}}\gamma_{1,s},$$

which gives

(A8) $$\frac{\alpha_{1}}{1+\frac{q_{2}}{p_{2}}\gamma_{2,s}}-\frac{\alpha_{1}}{1+\frac{q_{1}}{p_{1}}\gamma_{1,s}}<0.$$

Based on (A5), (A7), (A8), for $q_{1}>q_{2}$, if $\gamma_{1,s}\leq \gamma_{2,s}$, then Equation (A4) is impossible to be satisfied. Thus, for $q_{1}>q_{2}$, we $\gamma_{1,s}>\gamma_{2,s}$, which proves Lemma 2.

## Appendix C. Proof of Lemma 3

When *q* is a large number, i.e., $q\in [q_{1},\infty )$, the SINR constraint can be ignored, and thus the optimal received power of the IoT user is $p=\bar{P}$.

Substituting $q_{1}$, $q_{2}$, $\gamma_{1,s}$, $\gamma_{2,s}$ into (A3) and subtracting the two equations yields

(A9) $$\begin{array}{c} \frac{\alpha_{2}}{1+\gamma_{2,s}}-\frac{\alpha_{2}}{1+\gamma_{1,s}}+\frac{\alpha_{1}}{1+\frac{q_{2}}{\bar{P}}\gamma_{2,s}}-\frac{\alpha_{1}}{1+\frac{q_{1}}{\bar{P}}\gamma_{1,s}}=0. \end{array}$$

For $q_{1}>q_{2}$, if we assume $\gamma_{1,s}\geq \gamma_{2,s}$, then, we have

(A10) $$\frac{\alpha_{2}}{1+\gamma_{2,s}}-\frac{\alpha_{2}}{1+\gamma_{1,s}}\geq 0,$$

and

(A11) $$\frac{\alpha_{1}}{1+\frac{q_{2}}{\bar{P}}\gamma_{2,s}}-\frac{\alpha_{1}}{1+\frac{q_{1}}{\bar{P}}\gamma_{1,s}}\geq 0.$$

Based on (A10) and (A11), for $q_{1}>q_{2}$, if $\gamma_{1,s}\geq \gamma_{2,s}$, then (A9) is impossible to be satisfied. Thus, for $q_{1}>q_{2}$, we $\gamma_{1,s}<\gamma_{2,s}$, which proves Lemma 3.
